# Nutritional and metabolic process of the dung beetle *Phelotrupes auratus* depends on the plant ingredients that the herbivores eat

**DOI:** 10.1186/s12864-022-08982-y

**Published:** 2022-11-12

**Authors:** Takuma Sakamoto, Shun Sinzeki, Shunsuke Kakinuma, Eri Ishihara, Hiroko Tabunoki

**Affiliations:** 1 Institute of Global Innovation Research, Tokyo University of Agriculture and Technology, 3-5-8 Saiwai-Cho, Tokyo, Fuchu 183-8509 Japan; 2 Department of Science of Biological Production, Graduate School of Agriculture, Tokyo University of Agriculture and Technology, Tokyo, Japan; 3 Cooperative Major in Advanced Health Science, Graduate School of Bio-Applications and System Engineering, Tokyo University of Agriculture and Technology, Tokyo, Fuchu 183-8509 Japan; 4 Technology Research & Innovation, BIPROGY Inc, 1-1-1 Toyosu, Koto-Ku, Tokyo, 135-8560 Japan

**Keywords:** Dung beetle, Phelotrupes auratus, Nutritional and metabolism process, Transcriptome analysis, Genomic analysis, Sustainable land health cycle, Feeding habit

## Abstract

**Background:**

The dung beetle *Phelotrupes auratus* is a holometabolous insect belonging to the order Coleoptera, and it is widely distributed in Japan. The *P. auratus* habitat depends on herbivores. *P. auratus* eats the dung of the herbivores and carries it underground for its young. In this process, herbivore droppings disappear from the ground, not only keeping the ground hygienic but also maintaining good soil conditions for plant growth. In this way, a rich ecosystem is maintained. In recent years, the population of *P. auratus* has decreased, and the main cause has been the decrease in grazing land. It seems that Japanese dung beetles are mainly dependent on herbivores for nutrient sources. However, the physiological relationship between herbivores and *P. auratus* has not been well investigated. Here, we investigated the nutritional metabolism system of *P. auratus* by performing whole gene expression analysis of individuals collected from two areas where the ecosystem is occupied by different herbivores.

**Results:**

We obtained 54,635 transcripts from *P. auratus* from Nara Park and Cape Toi and identified 2,592 differentially expressed genes in the fat bodies of the Nara Park and Cape Toi groups. We annotated *P. auratus* transcripts using *Homo sapiens* and *Drosophila melanogaster* genes as references; 50.5% of *P. auratus* transcripts were assigned to *H. sapiens* genes, and 54.0% of *P. auratus* transcripts were assigned to *D. melanogaster* genes. To perform gene set enrichment analysis, we chose *H. sapiens* genes for *P. auratus* transcript annotation. Principal component analysis and gene set enrichment analysis revealed that the nutritional metabolism of *P. auratus* from Cape Toi might differ from that of *P. auratus* from Nara Park.

**Conclusion:**

We analyzed the nutritional metabolism system of *P. auratus* from Cape Toi and Nara Park and found that the characteristics of the nutritional metabolism process might depend on the plants consumed by the herbivores. Our findings will contribute to elucidating the relationships among habitat plants, herbivores, and dung decomposers and may aid in the maintenance of sustainable land health cycles.

**Supplementary Information:**

The online version contains supplementary material available at 10.1186/s12864-022-08982-y.

## Background

The dung beetles belong to the Coleopteran insect group. These insects primarily eat the droppings of mammals [1]. The dung beetles ship the droppings underground accompanying reproductive activity. The droppings disappear from the ground, keeping the environment clean [2]. The activity of the dung beetles gives air permeability and nutrients to the soil [3]. Therefore, dung beetles have an essential role in ecosystems. There are more than 159 species of dung beetles in Japan [4]. The dung beetle *Phelotrupes auratus* (Motschulsky 1857) is distributed nationwide in Japan and exists in several different metallic body colors, namely, red, green, and indigo blue [5]. *P. auratus* is a coprophagous insect that mainly eats the droppings of herbivores, and their habitat depends on herbivores [6]. *P. auratus* not only eats the droppings of the herbivores but also carries them underground for its young [3]. Thereby *P. auratus* is categorized into paracoprid (Tunnelers) [6]. In this process, herbivore droppings are removed from the ground surface [7, 8]. Moreover, the ground is moderately tilled, allowing air and nutrients to circulate in the ground, supporting growing plant abundance and maintaining biodiversity [9–11]. *P. auratus* thereby maintains hygiene on the ground and contributes to good soil conditions for plant growth [12]. In this way, a rich ecosystem can be maintained. In recent years, the grazing land area for domestic animals has decreased, and the populations of many Japanese dung beetles have also decreased [10–12]. It seems that Japanese dung beetles are mainly dependent on herbivores for nutrient sources. However, the physiological relationship between these herbivores and *P. auratus* has not been well investigated.

**Fig. 1:**
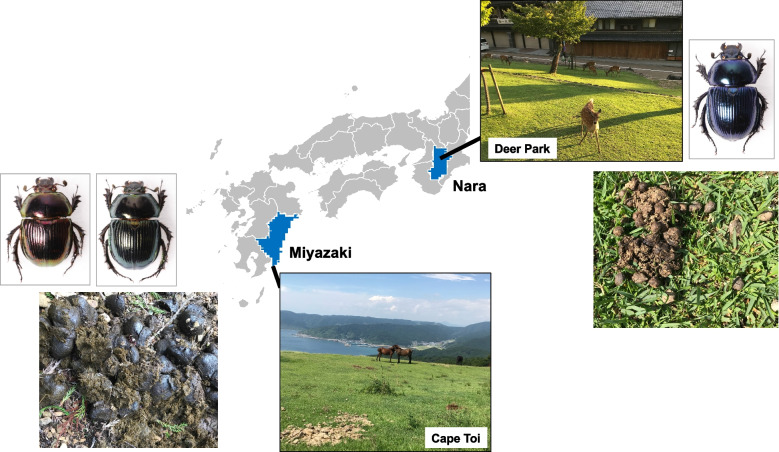
Sampling sites of *Phelotrupes auratus* in this study. Cape Toi is located in Miyazaki Prefecture, and Nara Park is located in Nara Prefecture. Lazuline *P. auratus* were collected from Nara Park, and green and red *P. auratus* were collected from Cape Toi

Several locations in Japan are predominantly occupied by particular herbivores, such as deer or horses. One is Nara Park, located in Nara Prefecture, Japan (Fig. 1). Approximately 1,300 Nara deer (*Cervus nippon,* also called Shin-roku in Japanese) live in this area [13]. Nara Park is associated with the Kasuga Shrine. According to KOJIKI [14], the god Heavenly-Deer-Deity gave Brave-Awful-Possessing-Male-Deity instructions to protect the country and keep peace across the land. This is the origin story of Shin-Roku, the Nara deer, and Nara deer have been considered to be sacred messengers. The god Heavenly-Deer-Deity (Nara deer) appears in the "Kasuga deer mandala" scroll [15]. Nara deer have been carefully maintained as sacred animals in Nara Park for more than 1200 years, since the Heian period [14].

The other location is Misaki Maki (currently called Cape Toi), located at the southernmost tip of Miyazaki prefecture. The Cape Toi Misaki ranch in the Fukushima region (currently Kushima City) was founded by the Akizuki feudal lord in 1697 for the purpose of replenishing war horses, and the Maki-union took over the operation when the feudal domains were abolished and prefectures were established in 1871 [16]. Wild horses (also called Misaki horses) are maintained here by Maki-kumiai. Misaki horses are bred naturally with year-round grazing and became one of the existing Japanese native horses designated as a protected species in 1953 [16]. Thus, the Misaki horse has been maintained and managed at Cape Toi for 300 years [16] and also occupies most of the ecosystem in Cape Toi. Against this historical background, particular herbivores, such as deer or horses, and relatively large areas of short-grass-type grasslands remain maintained in Nara Park and Cape Toi. Such ecological environments are valuable in the world.

Therefore, *P. auratus* and these herbivores have maintained a symbiotic relationship. The vegetation in Nara Park and Cape Toi is not identical. To date, the symbiotic relationships between herbivores and *P. auratus*, particularly focusing on nutritional metabolic systems, have not been investigated.

In this study*,* we investigated the nutritional and metabolism process of *P. auratus* by transcriptome analysis in two areas where the ecosystem is occupied by particular herbivores such as deer or horses.

## Results

### Survey of herbivores feed plants

To examine the forage plants that are eaten by herbivores, we performed a literature survey to compare the types of forage plants in Nara Park and Cape Toi. Nara deer mainly fed on the following plants: *Zoysia japonica Steud.*, *Hydrocotyle sibthorpioides*, *Paspalum thunbergia*, *Carex nervata Franch. et Sav.*, and *Trifolium repens* during the daytime in Nara Park in September (Table 1) [13]. Among the plant species, *Quercus myrsinifolia* and *H. sibthorpioides* are classified as medicinal plants [17]. *Q. myrsinifolia* has quercetin and kaempferol as flavonoids and pyrogallol and gallic acid as phenol derivatives [17]. *H. sibthorpioides* has chelidonine, protopine, and chelidimerine as alkaloids [18].

**Table 1:** Food plants for Misaki horses and Nara deer from July to September

Nara deer	Misaki horses
Castanopsis	*Zoysia japonica Steud*
*Quercus glauca*	*Paspalum thunbergii Kunth ex Steud*
*Quercus myrsinifolia*	*Imperata cylindrica*
*Pinus thunbergia*	*Rubus sieboldii*
*Cryptomeria japonica*	*Oplismenus undulatifolius*
*Zelkova serrata*	*Miscanthus sinensis*
*Cinnamomum camphora*	
*Zoysia japonica Steud*	
*Hydrocotyle sibthorpioides*	
*Paspalum thunbergia*	
*Carex nervata Franch. et Sav*	
*Trifolium repens*	

Meanwhile, Misaki horses mainly fed on several kinds of plants, such as *Zoysia japonica Steud.*, *Paspalum thunbergii Kunth ex Steud.*, *Imperata cylindrica*, and *Rubus sieboldii,* during the daytime in Cape Toi in September (Table 1) [19]. *I. cylindrica* is classified as a medicinal plant and is used following Japanese Pharmacopoeia [20]. *I. cylindrica* has cylinsrin, arundoin, and fernenol as triterpene [20]. The literature survey showed that each herbivores prefers to eat several kinds of plants and that they consume different kinds of plants based on their environments.

### Assignment of genes in the *P. auratus* genome sequence

We analyzed the *P. auratus* genome sequences from two individuals from Cape Toi (SRA accession numbers: DRR361505, DRR361506), and then the nucleotide sequences were assembled (Supplementary Table 1). Assembled genome sequences were evaluated by BUSCO (Supplementary Fig. 1). The graph showed that sample 1 included more duplicated, fragmented, and missing contigs than sample 2. Additionally, the maximum contig length of sample 2 was longer than that of sample 1 (Supplementary Table 1). The predicted genome size was 1.6 GB for sample 1 and 1.08 GB for sample 2. Sample 2 was also less duplicated and fragmented and was missing fewer contigs than sample 1. The genome size of another coleopteran insect, *Altica viridicyanea*, is approximately 864.8 Mb [21]. Thus, we chose to use the genome sequence from sample 2 for transcript assignment.

Next, we mapped the transcriptome data to the genome sequence and calculated the mapping rate. We performed RNA-Seq analysis on six Nara Park midgut samples (SRA accession numbers: DRR357601, DRR357602, DRR357603, DRR357607, DRR357608 and DRR357609), six Nara Park fat body samples (DRR357604, DRR357605, DRR357606, DRR357610, DRR357611, and DRR357612), six Cape Toi midgut samples (DRR357589, DRR357590, DRR357589, DRR357592, DRR357593, and DRR357594), and six Cape Toi fat body samples (DRR357595, DRR357596, DRR357597, DRR357598, DRR357599, and DRR357600). We confirmed that over 90% of transcripts were mapped to the genome sequence (Supplementary Table 2). Next, we counted the number of transcripts using the data of midgut and fat body transcripts. The estimated transcripts were 54,635. Then, we predicted the number of genes using the transcripts data, and the total number of genes was estimated to be 29,123. For the purpose of gene function annotation, we examined the homologous gene sequences in *H. sapiens* and *Drosophila melanogaster*. A total of 27,589 (50.5%) of the *P. auratus* genes had homology to the human genes. A total of 29,529 (54.0%) of the *P. auratus* genes had homology to *D. melanogaster* genes. Thus, we chose the *H. sapiens* gene sequence for *P. auratus* functional gene annotation.

### Identification of differentially expressed transcripts between different communities

**Fig. 2:**
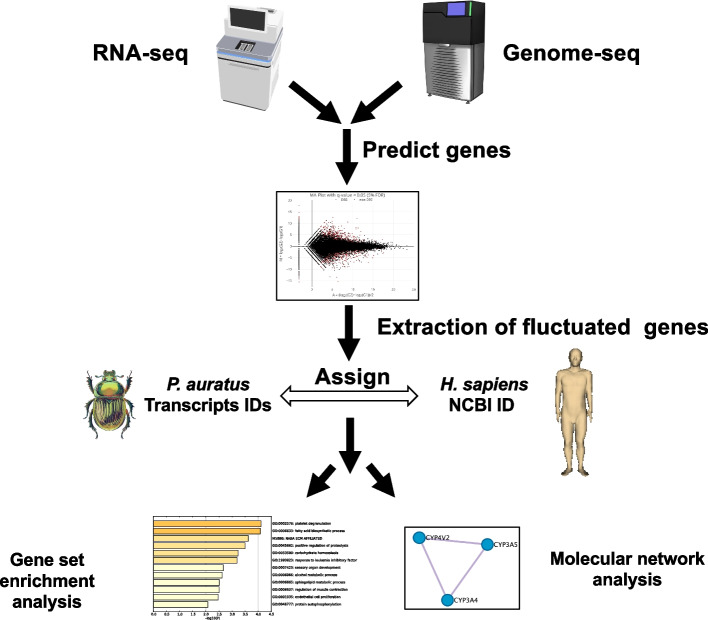
*Phelotrupes auratus* transcript analysis strategy. We performed genome sequencing and transcriptome analysis, followed by gene prediction. blastx was performed between *P. auratus* transcripts and human proteins in public databases to annotate the genes that were identified through RNA-Seq as having differential expression between Nara Park and Cape Toi samples. Then, we converted the transcript IDs from the *P. auratus* RNA-Seq to human NCBI IDs and constructed an assignment table. Finally, we performed gene enrichment analysis and molecular network analysis using the public database and determined molecular interactions in polyembryogenesis. The illustrations of experimental tools, humans and machines (https://togotv.dbcls.jp/ja/pics.html) are licensed under Creative Commons Attribution 4.0 International License (CC BY 4.0) (http://creativecommons.org/licenses/by/4.0/deed.en)

**Fig. 3:**
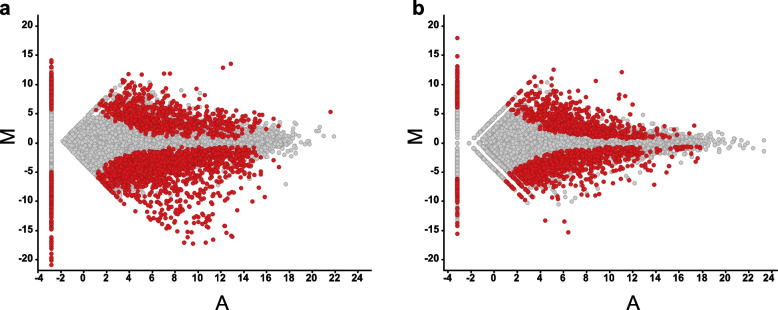
The extraction of differentially expressed transcripts between Nara Park and Cape Toi. Differentially expressed transcripts were extracted and plotted on the graph. Red dots indicate fluctuated transcripts with a false discovery rate, FDR < 0.05 between Nara Park and Cape Toi. The samples, DRR357601, DRR357602, DRR357603, DRR357592, DRR357593, DRR357594, DRR357604, DRR357605, DRR357606, DRR357598, DRR357599, and DRR357600 were used in the MA plot. M: Log_2_ (Cape Toi)—Log_2_ (Nara Park), A: (Log_2_ (Cape Toi) + Log_2_ (Nara Park))/2, **a**: Midgut samples, **b**: fat body samples

We constructed a procedure for functional gene annotation of *P. auratus* (Fig. 2). To compare the transcripts expression of each sample, the differentially expressed transcripts (false discovery rate, FDR < 0.05) of the midgut and fat body samples derived from Nara Park and Cape Toi were extracted using an MA plot (Fig. 3). The MA plot showed that there were 2,592 differentially expressed transcripts in the fat bodies between *P. auratus* derived from Cape Toi and those from Nara Park (Fig. 3a, Supplementary table 3). Additionally, the MA plot showed that there were 1,760 differentially expressed transcripts in the midgut between *P. auratus* derived from Cape Toi and those from Nara Park (Fig. 3b, Supplementary table 4).

In contrast, the MA plot showed that there were 30 (111) differentially expressed transcripts in the comparison of the fat body (midgut) of different *P. auratus* derived from Cape Toi (Supplementary Fig. 2a and b). The MA plot showed that there were 902 (491) differentially expressed transcripts in the comparison between the fat body (midgut) of different *P. auratus* derived from Nara Park. (Supplementary Fig. 3a and b). Fluctuations in transcript expression were greater in comparisons between individuals from different communities than among individuals from the same community, and the expression of these transcripts fluctuated more in the fat body than in the midgut.

### Principal component analysis and gene set enrichment analysis

**Fig. 4:**
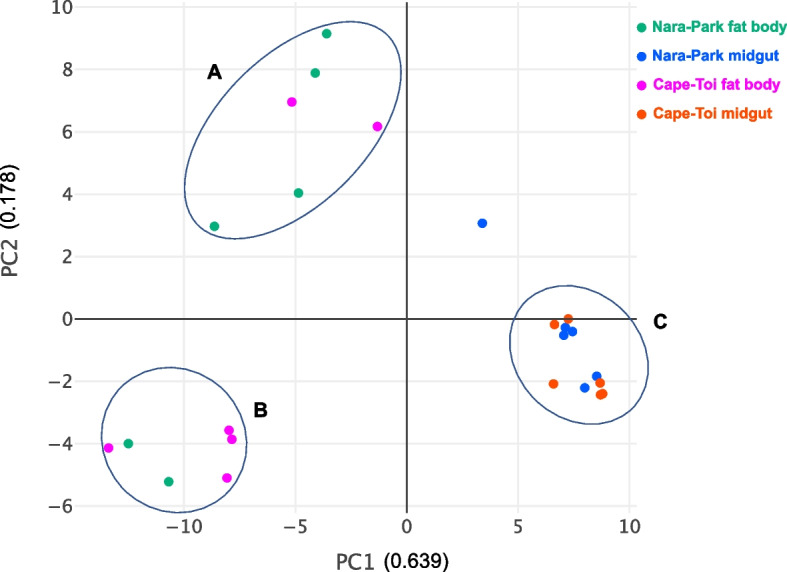
Principal component analysis (PCA) between Nara Park and Cape Toi transcriptomes. Green colored dots indicate Nara Park fat body transcripts, blue indicate Nara Park midgut transcripts, pink indicate Cape Toi fat body transcripts, and orange indicate Cape Toi midgut transcripts. The transcripts were divided into three groups: A, B, and C

Principal component analysis (PCA) was performed using R software to investigate the characteristics of the transcripts, and the transcripts were categorized into three groups (Fig. 4). Fat bodies derived from Nara Park were mainly distributed in group "A", fat bodies derived from Cape Toi were mainly distributed in group "B", and the midguts derived from both Nara Park and Cape Toi were distributed in group "C" (Fig. 4).

**Fig. 5:**
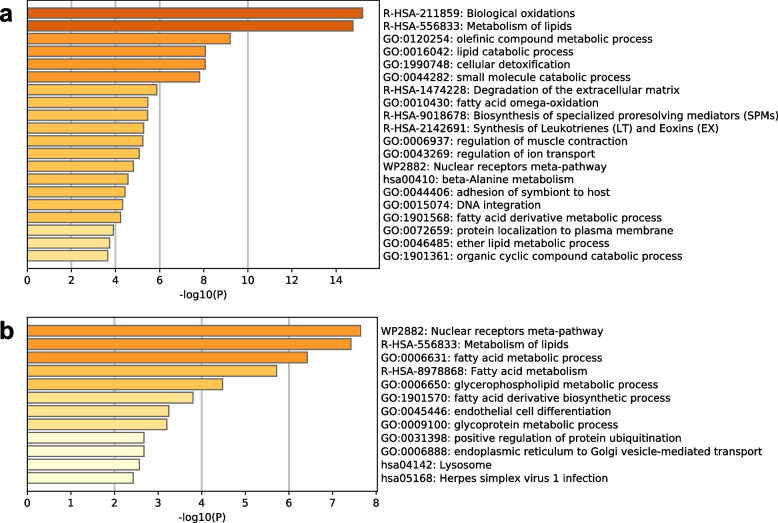
Gene enrichment analysis of differentially expressed fat body transcripts using Metascape. A bar graph for enriched terms across the input transcript lists; different colored bars indicate different *P* values. **a** Upregulated genes in the Cape Toi group (*n* = 6); **b** Upregulated genes in the Nara Park group (*n* = 6)

**Fig. 6:**
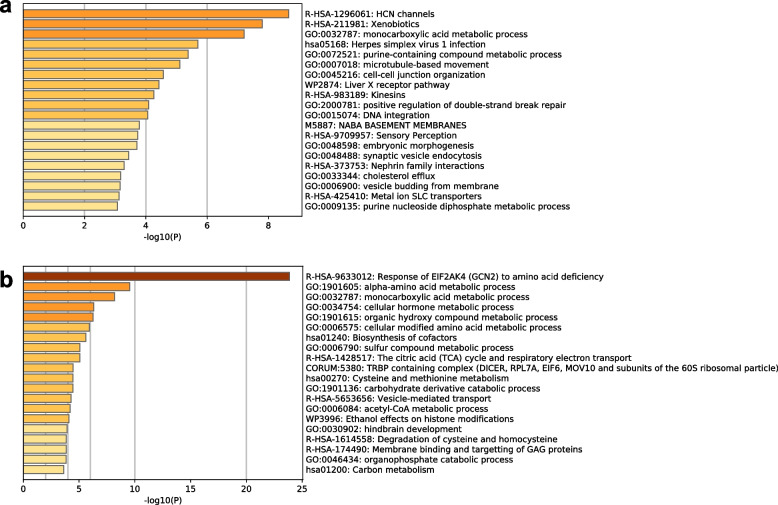
Gene enrichment analysis of differentially expressed midgut transcripts using Metascape. A bar graph for enriched terms across the input transcript lists; different colored bars indicate different *P* values. **a** Upregulated genes in the Cape Toi group (*n* = 6); **b** Upregulated genes in the Nara Park group (*n* = 6)

Next, we performed gene set enrichment analysis to characterize the upregulated transcripts involved in these groups. The transcripts from the fat bodies of Cape Toi-derived beetles were enriched in biological oxidation (R-HSA-211859) and lipid metabolism (R-HSA-556833) (Fig. 5a). The transcripts of fat bodies from Nara Park-derived beetles were enriched in the nuclear receptor meta-pathway (WP2882) and metabolism of lipids (R-HAS-556833) (Fig. 5b). The midgut transcripts of Cape Toi-derived beetles were enriched in HCN channels (R-HSA-1296061) and xenobiotics (R-HSA-211981) (Fig. 6a). The midgut transcripts of Nara Park-derived beetles were enriched in the response of EIF2AK4 (GCN2) to amino acid deficiency (R-HSA-9633012) (Fig. 6b). Gene set enrichment analysis of the fat body transcripts and midgut transcripts from the same Cape Toi community did not identify any significantly related GO terms (Supplementary Fig. 4a and b). Gene set enrichment analysis of the fat body transcripts from the same Nara Park community also did not identify any significantly related GO terms (Supplementary Fig. 5a and b). However, the midgut transcripts from the same Cape Toi community were involved in eukaryotic translation elongation (R-HSA-156842) and transport of small molecules (R-HAS-382551) (Supplementary Fig. 6a and b).

### Verification of gene expression related to biological oxidation

**Fig. 7:**
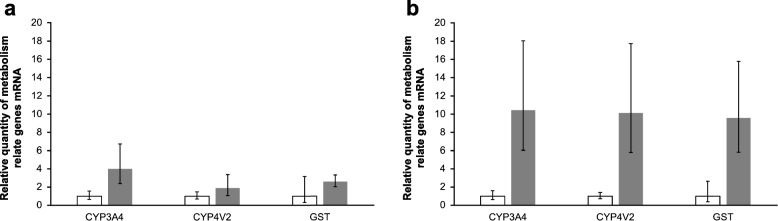
Validation of metabolism-related gene mRNA expression. Midgut (**a**) and fat body (**b**) samples of *P. auratus* from Nara Park and Cape Toi (*n* = 3 each) were used for RT–qPCR. mRNA expression levels in the midgut and fat body are presented as RQ values, which reflect the relative expression levels calculated using Nara Park samples as 1. Error bars represent the minimum/maximum expression levels relative to the mean RQ expression level. Ribosomal protein 6 was used as an endogenous control. White bars represent Nara Park samples, and gray bars represent Cape Toi samples

Gene set enrichment analysis suggested that the fat body transcripts of Cape Toi-derived animals were involved in biological oxidation (R-HSA-211859) [22]. This process is conserved in *D. melanogaster* (R-DME-211859) [23]. The *D. melanogaster* biological oxidation process is constituted by two phases: Phase I—functionalization of compounds, and Phase II—conjugation of compounds [23]. Thus, we considered in detail the genes that constructed these biological oxidation processes, and we identified Cytochrome P450 Family 3 Subfamily A Members (CYP3A), Cytochrome P450 Family 4 Subfamily V Members (CYP4V), and glutathione S-transferases (GSTs) as upregulated transcripts in Cape Toi fat body samples (Supplementary Tables 3, and 4). Next, we validated whether these transcripts were actually upregulated. We found that CYP3As, CYP4Vs, and GSTs were upregulated in Cape Toi fat body samples compared to Nara Park fat body samples (Fig. 7a, Supplementary Tables 5 and 6). Next, we analyzed the relationships of the factors encoded by these transcripts. We found that CYP3As and CYP4Vs were involved in phase I metabolism, while GSTs were involved in phase II metabolism (Fig. 7b).

## Discussion

In this study, we examined the *P. auratus* nutritional and metabolism system by transcriptome analysis in two areas where the ecosystem is occupied by particular herbivores. The Japanese *P. auratus* can be divided into two groups based on mitochondrial DNA haplotypes: Hokkaido, Honshu, and Shikoku are categorized into group A, and Kyushu and Yakushima are categorized into group B [5]. The *P. auratus* derived from Cape Toi are red or green in color and are classified into Group B. *P. auratus* from Nara Park has a indigo blue and is classified into Group A [5]. Thus, although *P. auratus* has geographical variations in color, the relationships between these mitochondrial DNA haplotypes and the exoskeleton body color remain unclear [24]. *P. auratus* is a coleopteran insect that mainly eats herbivores droppings, and their habitat depends on their herbivores [7]. *P. auratus* is widely distributed in Japan, from Hokkaido to Kyushu, in places where herbivores droppings occur [25].

*P. auratus* and its related herbivores have a symbiotic relationship. The deer or horses, as particular herbivores, are maintained in the Nara Park or Cape Toi by historical background [14, 16]. In other regions, it is challenging to determine the species that produces the droppings *P. aureus* ate. Still, it is easy to predict the species from which *P. aureus* ate droppings in Nara Park and Cape Toi. Thus, Nara park Cape Toi allows us to examine the relationship between *P. auratus* and the metabolites of deer or horses. Particular herbivores, such as deer or horses, have become the majority primary consumers in these ecosystems [13, 19]. Therefore, Nara Park and Cape Toi are excellent model environments for studying the nutritional and metabolism interaction among plants, primary consumers, and decomposers in an ecological community.

Deer and horses eat multiple plants that grow in their environment. A part of the plants ingested as food is absorbed through the metabolism of the herbivore, and another part is excreted as droppings [26]. Additionally, intestinal bacteria may be responsible for digesting cellulose and lignin contained in plants that are difficult to digest by the herbivore itself [26, 27]. In this way, horses and deer commonly feed on multiple species of herbaceous plants, and the digestion of cellulose depends in part on the intestinal flora. Therefore, it might be considered that both herbivores have similar physiological metabolic mechanisms.

PCA showed that the fat body transcript profiles of *P. auratus* derived from Nara Park and Cape Toi were separated from each other. In contrast, the midgut transcripts of *P. auratus* from these regions were not separated. These findings suggested that *P. auratus* nutritional metabolism may depend on the fat body rather than the midgut.

Next, we performed gene set enrichment analysis of the differentially expressed genes in the fat body and midgut of *P. auratus* and compared them with each other. We found that the cytochrome P450 3A4 isoform 1 (CYP3A4, NCBI ID: NP_059488), cytochrome P450 4V2 (CYP4V2, NCBI ID: NP_997235), and glutathione S-transferase theta-1 (GSTT1, NCBI ID: NP_000844) genes were upregulated in the fat body of Cape Toi *P. auratus*. These genes are involved in biological oxidations (R-HSA-211859) [22]. The pathway for the biological oxidants relates to the metabolism of foreign chemicals. Toxins from plants are included as foreign chemicals. These pathway processes are constituted by the functionalization of foreign chemicals (phase I), conjugation (phase II), and hydrophilic. The foreign chemicals are metabolized through this process and excreted from the body. CYPs play a role in the functionalization of a compound in this process. CYP3A4 is involved in the metabolism of fat-soluble compounds (GeneCards ID: GC07M099759) [28]. In particular, CYP3A4 metabolizes acetaminophen, codeine, cyclosporin A, diazepam, erythromycin, chloroquine, and some steroids and carcinogens. CYP4V2 is involved in oxidizing various substrates in the metabolic pathway that metabolizes fatty acid precursors into n-3 polyunsaturated fatty acids (GeneCards ID: GC04P186191) [29]. GSTs play a role in the conjugation of a compound in this process. GSTT1 catalyzes the conjugation of reduced glutathione to a variety of electrophilic and hydrophobic compounds (GeneCards ID: GC22Mi00270) [30]. These metabolic processes occur in the fat body [23]. Validation of the expression changes in these transcripts showed that the expression of these genes in the fat body was increased tenfold in the Cape Toi *P. auratus* compared with the Nara Park *P. auratus*.

On the other hand, no significant variability was observed in the expression of these transcripts in the midgut and fat body among *P. auratus* collected from the same region.

Considering the main food plants of Nara deer and Misaki horses during the day, *Zoysia japonica Steud.* was the main feed for both Nara deer and Misaki horses (Table 1). However, Nara deer typically return to the mountains in the evening, and they also consume tree leaves as food (Table 1) [8, 12]. There are two medicinal plants, *Q. myrsinifolia* and *H. sibthorpioides,* in Nara Park that contain flavonoids, phenol derivatives, and alkaloids. These chemicals have biological activities for animals [17, 18]. However, we did not find their effects in the transcriptome analysis in *P. auratus* collected from Nara Park. Probably, the droppings might not include these chemicals when *P. auratus* ate them. Thus, deer from Nara Park may not have eaten these plants at that time. In contrast, Misaki horses feed well on *Imperata cylindrica* in addition to *Zoysia japonica Steud. Imperata cylindrica* is classified as a gramineous plant and contains the herbal ingredients cylindrin, arundoin, and fernenol [20] and secondary plant metabolites such as 2,4-dihydroxy-6,4’-dimethoxychalcone, which are allelopathic ingredients [31]. These ingredients are mainly constituted by triterpenes, which are fat-soluble compounds [20]. The expression of CYP3A4, CYP4V2, and GSTT1 transcripts in the fat body was increased in *P. auratus* from Cape Toi compared with those from Nara Park. Therefore, it is speculated that the plant-derived secondary metabolites ingested by the herbivores might affect the nutritional and metabolism of *P. auratus*. It is inferred that the *P. auratus* nutritional and metabolism system may depend on the herbivores.

**Fig. 8:**
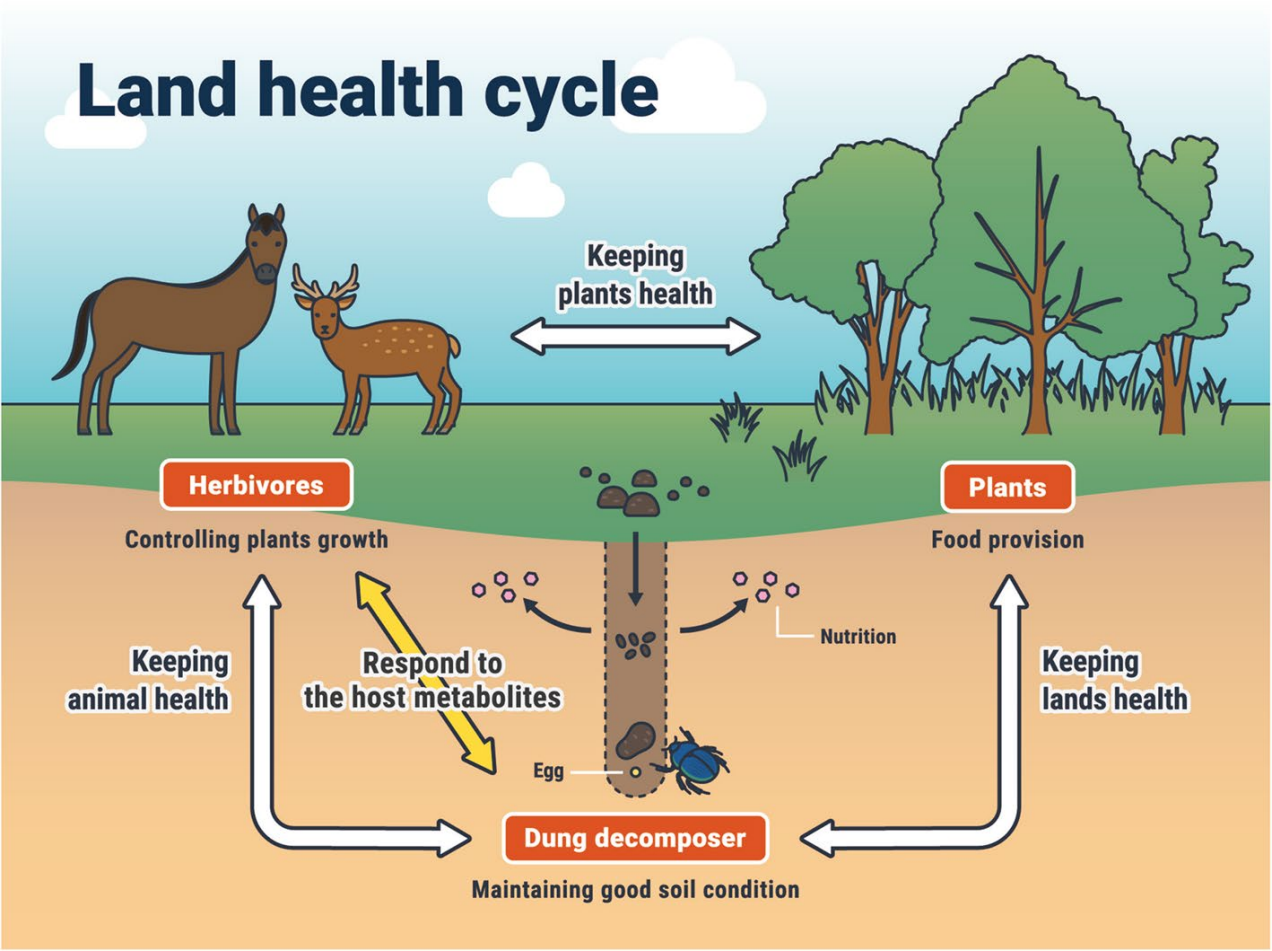
Plants, herbivores, and dung decomposers play a role in the sustainable land health cycle

Grasses are tramped every day by Misaki horses in Cape Toi or Nara deer in Nara Park [13, 19], and these grasses are eaten by Misaki horses or Nara deer and then excreted as droppings. The droppings are consumed by *P. auratus* and other dung beetles from spring to autumn [32]. At the same time, these droppings are carried deep into the ground to feed their larvae and thereby disappear from the ground surface [12]. This cycle not only keeps the land surface clean but also promotes good nutrient and air permeability in the soil and maintains the environmental microorganisms [2, 3]. It is presumed that such interactions among organisms enabled long-term sustainable ecosystem cycles to be built in Nara Park and Cape Toi (Fig. 8). Our findings suggest that the condition of the plants ingested by herbivores may also affect the growth of dung beetles that decompose their droppings. Therefore, habitat plants, herbivores, and dung decomposers maintain the land health cycle through their ecosystem balance.

Recently, livestock has been given medications to keep them healthy [33]. Livestock medications might also affect the growth and population maintenance of dung beetles. These cases suggest that the balance of the ecosystem collapses when human manipulation occurs. Hence, we must carefully consider the balance in the biological interaction to maintain the land health cycle.

In this study, we analyzed the nutritional and metabolism system of *P. auratus* from Cape Toi and Nara Park, choosing two regions that are mainly occupied by particular herbivores, such as deer or horses. Our findings suggest that the interaction between organisms is dependent on the balance of all organisms surrounding them. These interactions among organisms, as in Nara Park and Cape Toi, contribute to the creation and maintenance of biodiversity in these natural ecosystems. Our findings contribute to the elucidation of the relationships among habitat plants, herbivores, and dung decomposers and may aid in the construction of methods for maintaining sustainable land health cycles.

## Methods

### Insects

We obtained newly emerged *P. auratus* adults without scratch-wounding on their body surface from Cape Toi (Miyazaki Prefecture) and Nara Park (Nara Prefecture) with permission in 2019 and 2020. *P. auratus* new adults appear around September [32]. The permission numbers are 520–353 (Cape Toi) and 6–8 (Nara Park). Each *P. auratus* adult was transferred to a 50 mL tube (Cellstar CELLreactor 50 mL tube, #227,245, Greiner Bio-One Co. Ltd., Solingen Germany) with slightly wet paper and then shipped to the laboratory.

### Genome sequencing and analysis

We isolated DNA from the flight muscle of *P. auratus* adults (male and female, *n* = 1) of Cape Toi using a Blood & Cell Culture DNA Maxi Kit (Qiagen Co. Ltd., Valencia, CA, USA) according to the manufacturer’s instructions.

Then, we used an Agilent TapeStation 2200 (Agilent Technologies, Santa Clara, CA) to assess the DNA quality. Additionally, DNA libraries were constructed with 8 µg of DNA from these samples with a SMRTbell® Express Template Preparation Kit 2.0 (PacBio Co. Ltd. Menlo Park, CA., USA) according to the manufacturer’s instructions. Next, libraries were sequenced on the PacBio Sequel II platform, and subread files were generated. Next, subread files were assembled using Flye (ver. 2.8.3) [34]. The assembled genome sequences were checked using BUSCO (ver. 5.2.1) [35].

### RNA-Seq analysis

We isolated total RNA from the midgut and fat body in newly emerged *P. auratus* adults collected in Cape Toi (*n* = 6, three from red-colored beetles, and three from green colored beetles) or Nara Park (*n* = 6, three from beetles sampled in 2019, and three from beetles sampled in 2020) using a combination of TRIzol® LS Reagent and the PureLink® RNA Extraction Kit (Thermo Fisher Scientific Inc., Valencia, CA) according to the manufacturers’ instructions. Then, we used an Agilent TapeStation 2200 (Agilent Technologies, Santa Clara, CA) to assess the RNA quality. Additionally, cDNA libraries were constructed with 100 ng of total RNA from these samples with a TruSeq® Stranded mRNA Sample Preparation Kit (Illumina Inc., San Diego, CA) according to the manufacturer’s instructions. Next, the libraries were sequenced (100 bp, paired-end) on the Illumina NovaSeq6000 platform, and FASTQ files were assessed by Trim Galore! Version 0.4.5 (https://www.bioinformatics.babraham.ac.uk/projects/trim_galore/). The *P. auratus* genome sequence generated above was used as the reference genome sequence for the following data analysis. The obtained FASTQ sequence files were aligned to the reference draft genome asembly by HISAT2 with default parameters [36]. Next, SAM files were converted to BAM files using SAMtools v1.8 [37]. Using StringTie v1.3.4, the transcript abundance was estimated, and the count data were extracted. All statistical analyses were performed using R version 4.0.1 (https://www.r-project.org), the TCC package [38], and the DEseq2 package (version 1.30.1) [39]. We generated a scatter plot using TIBCO Spotfire Desktop v7.6.0 with the “Better World” program license (TIBCO Spot re, Inc., Palo Alto, CA; http://spotfire.tibco.com/better-world-donation-program/). The sequence data (FASTQ files) were deposited in the DDBJ Sequence Read Archive (Supplementary Table 7).

### Data visualization

A scatter plot with gene IDs were generated using TIBCO Spotfire Desktop version 7.6.0 (TIBCO Software Inc., Palo Alto, CA, USA) with TIBCO Software’s “Better World” program license. PCA was conducted using R software (version 4.0.4) with the TCC-GUI (version 1.0) method [40].

### *P. auratus* gene functional annotation pipeline for gene set enrichment analysis

The transcripts were extracted from the *P. auratus* genome using gffread software (version 0.12.1) (https://github.com/gpertea/gffread). To annotate the *P. auratus* genes, we identified genes homologous to those of humans by conducting a systematic BLAST (Version 2.12.0) search (blastx) with a cutoff E-value of significant similarity at 1e–10 (query: *P. auratus* cDNA sequence; database: whole human cDNA sequence set (GRCh38.p13) from the Ensembl database). Using the generated assignment table, we reconstructed conserved pathways common to *P. auratus* and humans by projecting *P. auratus* genes onto the human pathway. We converted the gene IDs from the *P. auratus* RNA-Seq data to *H. sapiens* NCBI RefSeq IDs using the assignment table. To obtain significant molecular interactions with corresponding *E* values, we performed gene set enrichment analysis using Metascape (Version 3.5; http://metascape.org/) [41]. The gene list for Metascape analysis was generated from the TCC output.

### Purification of total RNA and cDNA synthesis from midgut and fat body samples

Total RNA from the *P. auratus* midgut (*n* = 3) and fat body (*n* = 3) was isolated and purified. The midgut and fat body were separately homogenized with TRIzol™ Reagent (Life Technologies, Carlsbad, CA, USA) and processed for RNA purification in accordance with the manufacturer’s instructions. Total RNA (1 μg) was treated with amplification-grade DNase I (Life Technologies). Thereafter, 500 ng of DNase-treated total RNA was employed as a template for cDNA synthesis using the PrimeScript™ 1st strand cDNA Synthesis Kit (Takara Bio, Inc., Kusatsu, Shiga, Japan).

Real-time quantitative PCR (RT–qPCR) was performed in a 20 μL reaction volume containing 0.5 μL of cDNA template and specific primers (Table 2) with KAPA SYBR® FAST qPCR Kit Master Mix (2X) ABI Prism™ (Sigma–Aldrich Corporation, St. Louis, MO, USA), in accordance with the manufacturer’s instructions, on a StepOnePlus™ Real-Time PCR System (Applied Biosystems, Carlsbad, CA, USA). PCR thermal cycle was as follows: initial denaturation at 95 °C for 3 min, then 40 cycles at 95 °C for 3 s, and 60 °C for 20 s. Relative gene expression levels were calculated using the 2–∆∆Ct method, with the *P. auratus* ribosomal protein 6 gene as an endogenous reference for the standardization of RNA expression levels. All data were calibrated against universal reference data. Relative expression levels normalized to a reference sample are represented as relative quantification (RQ) values. All samples were assayed with three biological replications.

**Table 2:** Primers used in RT–qPCR

Gene name	Forward	Reverse
Ribosomal protein 6	5’-TGAACATTTCGTACCCCGCA-3’	5’-GGACACCTTGCTTCATGGGA-3’
CYP3A4	5’-GATGCCCTACCTCGACATGG-3’	5’-CTCTTGTCCCGGCTTCCAAT-3’
CYP4V2	5’-CGCTCAGACTTTTCCCGAGT-3’	5’-ATTTCGTGGCCCTGCACTAA-3’
GSTT1	5’-GCCGAATGTTACTACCCCGT-3’	5’-GAAACCAACTGCCTCTCCCA-3’

## Supplementary Information

**Additional file 1:** **Supplementary figure 1.** Assessment forassembled *P.auratus* genome sequences. **Supplementary figure 2.** The extraction of fluctuated transcriptsbetween green and red coloured beetles in the Cape-Toi group. Fluctuatedtranscripts were extracted and plotted on the graph. Red dots indicatefluctuated transcripts with a false discovery rate, FDR <0.05 between greenand red coloured beetles in the Cape-Toi group. The samples, DRR357589,DRR357590, DRR357591, DRR357592, DRR357593, DRR357594, DRR357595, DRR357596,DRR357597, DRR357598, DRR357599, and DRR357600, were used in the MA plot. M:Log_2_ (Red coloured beetles) - Log_2_ (Green coloured beetles),A: (Log_2_ (Red coloured beetles) + Log_2_ (Green coloured beetles))/2,(a) : midgut, (b) : fat body. **Supplementary figure 3.** Fluctuated transcripts wereextracted in the Nara-Park (Three frombeetles sampled in 2019, and three from beetles sampled in 2020).Fluctuated transcripts were extracted and plotted on the graph. Red dotsindicate fluctuated transcripts with a false discovery rate, FDR <0.05,between beetles sampled in 2019, and beetles sampled in2020. Thesamples, DRR357601, DRR357602, DRR357603, DRR357607, DRR357608, DRR357609,DRR357604, DRR357605, DRR357606, DRR357610, DRR357611, and DRR357612 were usedin MA plot. M: Log_2_ (2019 Nara Park) - Log_2_ (2020 NaraPark), A: (Log_2_ (2019 Nara Park) + Log_2_ (2020 Nara Park))/2,(a) : midgut, (b) : fat body. **Supplementary figure 4.** The gene enrichmentanalysis of fluctuated fat body transcripts in the Cape-Toi group usingMetascape. A bar graph for enriched terms across the input transcripts lists;different colored bars, P values. (a) Upregulated genes in the group a (*n*=3); (b)Upregulated genes in the group b (*n*=3). **Supplementaryfigure 5.** The gene enrichment analysis of fluctuated fat body transcripts inthe Nara-Park group using Metascape. A bar graph for enriched terms across theinput transcripts lists; different colored bars, P values. (a) Upregulatedgenes in the group a (*n*=3); (b) Upregulated genes in the group b (*n*=3). **Supplementary figure 6.** The gene enrichmentanalysis of fluctuated midgut transcripts in the Nara-Park group usingMetascape. A bar graph for enriched terms across the input transcripts lists;different colored bars, P values. (a) Upregulated genes in the group a (*n*=3);(b) Upregulated genes in the group b (*n*=3). **Supplementary Table 1.** Assembled genomesequence in *P. auratus. ***Supplementary Table 2.** Mapping rate foreach transcriptome data. **Supplementary Table 5.** The count data inthe midgut for the up-regulated genes in the biological oxidations. **SupplementaryTable 6.** The count data in the fat body for the up-regulated genes in thebiological oxidations. **Supplementary Table 7.** DDBJ SRA accessionnumbers for RNA seq in this study.

**Additional file 2:** **Supplementary Table 3.** Fluctuated transcript list in the midgut between Cape Toi and Nara Park.

**Additional file 3:** **Supplementary Table 4.** Fluctuated transcript list in the fat body between Cape Toi and Nara Park.

## Data Availability

The RNA-seq datasets analysed during the current study are available in the Sequence Read Archive (SRA) repository, DRR357601, DRR357602, DRR357603, DRR357607, DRR357608, DRR357609, DRR357604, DRR357605, DRR357606, DRR357610, DRR357611, DRR357612, DRR357589, DRR357590, DRR357589, DRR357592, DRR357593, DRR357594, DRR357595, DRR357596, DRR357597, DRR357598, DRR357599, and DRR357600. The genome datasets analysed during the current study are available in the Sequence Read Archive (SRA) repository, DRR361505 and DRR361506.

## Abbreviations

GST: Glutathione S-transferases
PCA: Principal component analysis
RQ: Relative quantification
SRA: Sequence Read Archive
