# Supplementary material for: Nutritional and metabolic process of the dung beetle Phelotrupes auratus depends on the plant ingredients that the herbivores eat
Source: BMC Genomics. 2022 Nov 12;23:751. doi: 10.1186/s12864-022-08982-y (PMC9655807; doi:10.1186/s12864-022-08982-y)
Supplement: Supplementary file 1 — Additional file 1: Supplementary figure 1. Assessment forassembled P.auratus genome sequences. Supplementary figure 2. The extraction of fluctuated transcriptsbetween green and red coloured beetles in the Cape-Toi group. Fluctuatedtranscripts were extracted and plotted on the graph. Red dots indicatefluctuated transcripts with a false discovery rate, FDR <0.05 between greenand red coloured beetles in the Cape-Toi group. The samples, DRR357589,DRR357590, DRR357591, DRR357592, DRR357593, DRR357594, DRR357595, DRR357596,DRR357597, DRR357598, DRR357599, and DRR357600, were used in the MA plot. M:Log2 (Red coloured beetles) - Log2 (Green coloured beetles),A: (Log2 (Red coloured beetles) + Log2 (Green coloured beetles))/2,(a) : midgut, (b) : fat body. Supplementary figure 3. Fluctuated transcripts wereextracted in the Nara-Park (Three frombeetles sampled in 2019, and three from beetles sampled in 2020).Fluctuated transcripts were extracted and plotted on the graph. Red dotsindicate fluctuated transcripts with a false discovery rate, FDR <0.05,between beetles sampled in 2019, and beetles sampled in2020. Thesamples, DRR357601, DRR357602, DRR357603, DRR357607, DRR357608, DRR357609,DRR357604, DRR357605, DRR357606, DRR357610, DRR357611, and DRR357612 were usedin MA plot. M: Log2 (2019 Nara Park) - Log2 (2020 NaraPark), A: (Log2 (2019 Nara Park) + Log2 (2020 Nara Park))/2,(a) : midgut, (b) : fat body. Supplementary figure 4. The gene enrichmentanalysis of fluctuated fat body transcripts in the Cape-Toi group usingMetascape. A bar graph for enriched terms across the input transcripts lists;different colored bars, P values. (a) Upregulated genes in the group a (n=3); (b)Upregulated genes in the group b (n=3). Supplementaryfigure 5. The gene enrichment analysis of fluctuated fat body transcripts inthe Nara-Park group using Metascape. A bar graph for enriched terms across theinput transcripts lists; different colored bars, P values. (a) Upregulatedgenes in the group a (n=3); (b) Up [file 12864_2022_8982_MOESM1_ESM.docx]

**Nutritional and metabolic process of the dung beetle *Phelotrupes auratus* depends on the plant ingredients that the herbivores eat**

Takuma Sakamoto^1,2^, Shun Sinzeki^2^, Shunsuke Kakinuma^3^, Eri Ishihara^4^, Hiroko Tabunoki^1,2,3*^

^1^ Institute of Global Innovation Research, Tokyo University of Agriculture and Technology, 3-5-8 Saiwai-cho, Fuchu, Tokyo, 183-8509, Japan.

^2^ Department of Science of Biological Production, Graduate School of Agriculture, Tokyo University of Agriculture and Technology, Tokyo, Japan.

^3^ Cooperative Major in Advanced Health Science, Graduate School of Bio-Applications and System Engineering, Tokyo University of Agriculture and Technology, Fuchu, Tokyo, 183-8509, Japan

^4^ Technology Research & Innovation, BIPROGY Inc. 1-1-1 Toyosu, Koto-ku, Tokyo, Japan 135-8560

Takuma Sakamoto and Shun Sinzeki contributed equally.

*Corresponding author: Hiroko Tabunoki

This PDF includes:

Supplementary Figure 1.

Supplementary Figure 2.

Supplementary Figure 3.

Supplementary Figure 4.

Supplementary Figure 5.

Supplementary Figure 6.

Supplementary Table 1.

Supplementary Table 2.

Supplementary Table 3.

Supplementary Table 4.

Supplementary Table 5.

Supplementary Table 6.

Supplementary Table 7.

Supplementary figure 1. Assessment for assembled *P.auratus* genome sequences.

Busco used for evaluation of genome assemble. 1: Genome sample from Cape Toi, individual1; 2: Genome sample from Cape Toi, individual 2. Each color indicates core gene sets in the graph.

Supplementary figure 2. The extraction of fluctuated transcripts between green and red coloured beetles in the Cape-Toi group. Fluctuated transcripts were extracted and plotted on the graph. Red dots indicate fluctuated transcripts with a false discovery rate, FDR <0.05 between green and red coloured beetles in the Cape-Toi group. The samples, DRR357589, DRR357590, DRR357591, DRR357592, DRR357593, DRR357594, DRR357595, DRR357596, DRR357597, DRR357598, DRR357599, and DRR357600, were used in the MA plot. M: Log_2_ (Red coloured beetles) - Log_2_ (Green coloured beetles), A: (Log_2_ (Red coloured beetles) + Log_2_ (Green coloured beetles))/2, (a) : midgut, (b) : fat body.

Supplementary figure 3. Fluctuated transcripts were extracted in the Nara-Park (Three from beetles sampled in 2019, and three from beetles sampled in 2020). Fluctuated transcripts were extracted and plotted on the graph. Red dots indicate fluctuated transcripts with a false discovery rate, FDR <0.05, between beetles sampled in 2019, and beetles sampled in 2020. The samples, DRR357601, DRR357602, DRR357603, DRR357607, DRR357608, DRR357609, DRR357604, DRR357605, DRR357606, DRR357610, DRR357611, and DRR357612 were used in MA plot. M: Log_2_ (2019 Nara Park) - Log_2_ (2020 Nara Park), A: (Log_2_ (2019 Nara Park) + Log_2_ (2020 Nara Park))/2, (a) : midgut, (b) : fat body.

Supplementary figure 4. The gene enrichment analysis of fluctuated fat body transcripts in the Cape-Toi group using Metascape. A bar graph for enriched terms across the input transcripts lists; different colored bars, P values. (a) Upregulated genes in the group a (n=3); (b) Upregulated genes in the group b (n=3).

Supplementary figure 5. The gene enrichment analysis of fluctuated fat body transcripts in the Nara-Park group using Metascape. A bar graph for enriched terms across the input transcripts lists; different colored bars, P values. (a) Upregulated genes in the group a (n=3); (b) Upregulated genes in the group b (n=3)

Supplementary figure 6. The gene enrichment analysis of fluctuated midgut transcripts in the Nara-Park group using Metascape. A bar graph for enriched terms across the input transcripts lists; different colored bars, P values. (a) Upregulated genes in the group a (n=3); (b) Upregulated genes in the group b (n=3)

Supplementary Table 1. Assembled genome sequence in *P. auratus*

| individual | Number of contig | Sum of contig length | Average of contig length | Maximum of contig length |
| --- | --- | --- | --- | --- |
| 1 | 36,291 | 1,577,576,937 | 43,470 | 1,908,500 |
| 2 | 19,932 | 1,066,449,501 | 53,504 | 7,365,120 |

Supplementary Table 2. Mapping rate for each transcriptome data.

| Mapping rate (%) in Nara Park | | Mapping rate (%) in Cape Toi | |
| --- | --- | --- | --- |
| Fat body | Midgut | Fat body | Midgut |
| 92.2 ± 0.004 | 91.1 ± 0.024 | 94.9 ± 0.017 | 92.0 ± 0.079 |

Mapping rate show as mean ± SD

Supplementary Table 5. The count data in the midgut for the up-regulated genes in the biological oxidations.

Supplementary Table 6. The count data in the fat body for the up-regulated genes in the biological oxidations.

Supplementary Table 7. DDBJ SRA accession numbers for RNA seq in this study

| Location | Sample information | Accession numbers | Collection year / body colour |
| --- | --- | --- | --- |
| Nara Park | Midgut | DRR357601 | 2020 / blue |
| Nara Park | Midgut | DRR357602 | 2020 / blue |
| Nara Park | Midgut | DRR357603 | 2020 / blue |
| Nara Park | Midgut | DRR357607 | 2019 / blue |
| Nara Park | Midgut | DRR357608 | 2019 / blue |
| Nara Park | Midgut | DRR357609 | 2019 / blue |
| Nara Park | Fat body | DRR357604 | 2020 / blue |
| Nara Park | Fat body | DRR357605 | 2020 / blue |
| Nara Park | Fat body | DRR357606 | 2020 / blue |
| Nara Park | Fat body | DRR357610 | 2019 / blue |
| Nara Park | Fat body | DRR357611 | 2019 / blue |
| Nara Park | Fat body | DRR357612 | 2019 / blue |
| Cape Toi | Midgut | DRR357589 | 2020 / green |
| Cape Toi | Midgut | DRR357590 | 2020 / green |
| Cape Toi | Midgut | DRR357591 | 2020 / green |
| Cape Toi | Midgut | DRR357592 | 2020 / red |
| Cape Toi | Midgut | DRR357593 | 2020 / red |
| Cape Toi | Midgut | DRR357594 | 2020 / red |
| Cape Toi | Fat body | DRR357595 | 2020 / green |
| Cape Toi | Fat body | DRR357596 | 2020 / green |
| Cape Toi | Fat body | DRR357597 | 2020 / green |
| Cape Toi | Fat body | DRR357598 | 2020 / red |
| Cape Toi | Fat body | DRR357599 | 2020 / red |
| Cape Toi | Fat body | DRR357600 | 2020 / red |
